# Combat phytopathogenic bacteria employing *Argirium*-*SUNCs*: limits and perspectives

**DOI:** 10.1007/s00253-024-13189-0

**Published:** 2024-06-01

**Authors:** Benedetta Orfei, Chiaraluce Moretti, Anna Scian, Michela Paglialunga, Stefania Loreti, Giuseppe Tatulli, Luca Scotti, Antonio Aceto, Roberto Buonaurio

**Affiliations:** 1 Department of Agricultural, Food and Environmental Sciences, University of Perugia, Perugia, Italy; 2 Research Centre for Plant Protection and Certification, Council for Agricultural Research and Economics (CREA), Rome, Italy; 3 Department of Medical, Oral and Biotechnological Sciences, “G. d’Annunzio” University of Chieti-Pescara, Chieti, Italy

**Keywords:** Silver nanoparticles, *Pseudomonas syringae* pv. tomato, *Xanthomonas vesicatoria*, *Xylella fastidiosa* subsp. *pauca*, *Clavibacter michiganensis*

## Abstract

**Abstract:**

Bacterial plant diseases are difficult to control as the durability of deployed control measures is thwarted by continuous and rapid changing of bacterial populations. Although application of copper compounds to plants is the most widespread and inexpensive control measure, it is often partially efficacious for the frequent appearance of copper-resistant bacterial strains and it is raising concerns for the harmful effects of copper on environment and human health. Consequently, European Community included copper compounds in the list of substances candidates for substitution. Nanotechnologies and the application of nanoparticles seem to respond to the need to find new very effective and durable measures. We believe that *Argirium*-*SUNCs*®, silver ultra nanoclusters with an average size of 1.79 nm and characterized by rare oxidative states (Ag^2+/3+^), represent a valid candidate as a nano-bactericide in the control of plant bacterial diseases. Respect to the many silver nanoparticles described in the literature, *Argirium*-*SUNCs* have many strengths due to the reproducibility of the synthesis method, the purity and the stability of the preparation, the very strong (less than 1 ppm) antimicrobial, and anti-biofilm activities. In this mini-review, we provide information on this nanomaterial and on the possible application in agriculture.

**Key points:**

*• Argirium-SUNCs have strong antimicrobial activities against phytopathogenic bacteria.*

*• Argirium-SUNCs are a possible plant protection product.*

*• Argirium-SUNCs protect tomato plants against bacterial speck disease.*

**Graphical Abstract:**

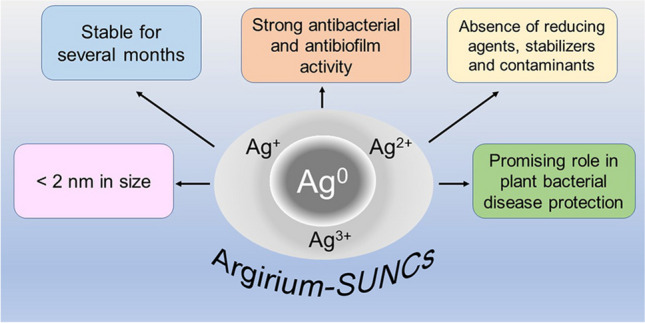

## Introduction

Plant diseases caused by phytopathogenic bacteria are a constant threat to crop production causing significant annual losses on a global scale, estimated of over 1 billion of dollars (Mansfield et al. 2012; Kannan et al. 2015). In addition, increased global movement of plant products and climate change are exacerbating this situation (Ristaino et al. 2021; IPPC Secretariat 2021). An array of control measures are deployed to combat plant bacterial diseases such as improved cultural practices; application of bactericides, plant activators, and biocontrol agents; and the use of resistant cultivars when available (Sharma et al. 2022). However, effective management of plant bacterial diseases remains a challenge as the durability of deployed control measures is thwarted by continuous and rapid changing of bacterial populations (Sharma et al. 2022). The main measure adopted worldwide for controlling plant bacterial diseases is the application of copper compounds, which are currently often only partially efficacious for the frequent appearance of copper-resistant bacterial strains (Sundin et al. 2016; Fan et al. 2022). Furthermore, the use of copper compounds at high dosages and application frequency has generated their accumulation in the soil and the contamination of surface and sub-surface water, raising concerns on their use in agriculture (Lamichhane et al. 2018). In fact, for these reasons, the European Commission has included copper compounds in the list of substances as candidates for substitution.

Moreover, the extensive and reiterated use of antibiotics (*e*.*g*., streptomycin, oxytetracycline, oxolinic acid, gentamicin) for controlling bacterial plant diseases, permitted in a number of countries but not in the European ones, has often induced the selection of resistance bacterial strains (evolved through the acquisition of a resistance determinant via horizontal gene transfer, presumably been acquired from non-pathogenic epiphytic bacteria cohabiting in plant hosts under antibiotic selection) (Sundin and Wang 2018). The same mechanism has driven the antibiotic resistance crisis currently affecting the human population (Perry and Wright 2013; Smillie et al. 2011).

Therefore, there is an increasing need to develop alternative solutions to manage plant bacterial diseases, which should be durable, sustainable, accessible to farmers, and environmentally friendly. Nanotechnology, recognized as the first of the “Key Enabling Technologies” by the European Commission in Horizon 2020, seems to meet this need (Xu et al. 2022). In fact, in the recent decade, nanotechnology and nanomaterials have received considerable attention in the agriculture field (Prasad et al. 2017; Saritha et al. 2022), plant bacterial disease protection included (Sundin et al. 2016; Elmer and White 2018; Balestra and Fortunati 2022; Sharma et al. 2022, 2023).

The recent results we obtained with the nanomaterial silver ultra nanoclusters (< 2 nm) (*Argirium*-*SUNCs*^®^), published in Applied Microbiology and Biotechnology (Orfei et al. 2023a), seem to open up new perspectives in the chemical control of plant bacterial diseases, which will be described in the present minireview.

## Silver ultra nano clusters (*Argirium*-*SUNCs*): properties and antibacterial applications

Among the nanomaterials used to control plant bacterial diseases, silver nanoparticles (AgNPs) are the most explored (Tariq et al. 2022), probably because they are widely used in medical field for their strong antibacterial activity even against multidrug-resistant human pathogenic bacteria (More et al. 2023). As for other nanomaterials, AgNPs can be synthesized by a plethora of chemical, physical, and biological protocols (Yaqoob et al. 2020), using top-down and bottom-up approaches (Shanmuganathan et al. 2019). Between the two approaches, the latter ones, which involve the production of nanoparticles atom-by-atom mainly via biological and chemical synthesis processes, can be preferred over top-down methods because they allow greater control over morphological characteristics such as the size and shape of the produced nanoparticles (Villaverde-Cantizano et al. 2021). Respect to the many AgNPs described in the literature, *Argirium*-*SUNCs* are synthetized by a high reproducible electrochemical protocol (Patent EP-18,181,873) (Scotti et al. 2017). In the last years, electrochemical synthesis of nanomaterials has proven to be a valid alternative to the main chemical synthesis methods because it allows greater control over morphological characteristics such as the size and shape of the produced nanoparticles using conventional systems and inexpensive reagents (Singaravelan and Bangaru Sudarsan Alwar 2015). Another advantage provided by this synthesis protocol is that stabilizing agents may not be necessary in the reaction in order to obtain a stable dispersion, cutting down additional costs in the production process (Khaydarov et al. 2009). Specifically, *Argirium*-*SUNCs* are prepared in ultra-pure water without reducing agents, stabilizers, and contaminants, which can interfere with efficacy and toxicity of the nanomaterials by altering the expected results (Siddiqi et al. 2018). The data published to date demonstrate the stability of the solutions at pH 2–12 and at temperatures 10–90 °C (Grande et al. 2020; Gasbarri et al. 2021). There are no published data on stability in enzymes, but they are currently in progress and will be the subject of a forthcoming publication.

In the core of the nanoparticle of *Argirium*-*SUNCs*, metallic Ag^0^ is present, while in the external shells, due to the electron-attracting action of the water oxygen atoms, Ag^+^, Ag^2+^, and Ag^3+^ silver oxides, never observed in a stable form before (Molina-Hernandez et al. 2023). This configuration ensures them a high anionic salvation surrounding of *SUNCs* (zeta potential > −50 mV) which explains their stability for several months in ultra-pure water solution without large aggregates, while the presence of silver oxides on the clusters surface explains their enhanced redox properties towards biological targets (Molina-Hernandez et al. 2023). As a consequence, *Argirium*-*SUNCs* are capable of a strong broad spectrum antimicrobial activity not only due to their size, but also to their atomic structure. In fact, at very low concentrations (< 1 ppm), *Argirium*-*SUNCs* show strong antimicrobial and antibiofilm activities against Gram-negative (*Pseudomonas syringae* pv. tomato, *Xanthomonas vesicatoria* and *Xylella fastidiosa*) and Gram-positive (*Clavibacter michiganensis*) phytopathogenic bacteria (Orfei et al. 2023a) as well as mammalian pathogenic ones (Pompilio et al. 2018), and common pathogenic and spoilage food bacteria (Mancusi et al. 2024), while, in general, the antibacterial activity of AgNPs ranges between 10 and 100 ppm (Duval et al. 2019) (Table 1).

**Table 1:** Activity in vitro of *Argirium-SUNCs* against phytopathogenic bacteria (in bold) in comparison with other silver nanoparticles

Average or range size (nm)	Zpuls value (mV)	Target bacteria	MIC (ppm)	References
**1.97**	**−50.00**	**Pst, Xv, Cm, Xf**	**0.13–0.82**	**Orfei et al. (**2023a**)**
8.27	−17.08	At, Pc, Ps, Xc	8–256	Trzcińska-Wencel et al. (2023b)
13.19	−11.50	Pc	8	Wei et al. (2022)
15.56	−38.43	At, Pc, Ps, Xc	8–128	Trzcińska-Wencel et al. (2023a)
16.50	−27.00	Xoo	7.5	Namburi et al. (2021)
27.00	−11.80	Xap, Rs	6.25–12.5	Vanti et al. (2020)
35.00	−48.00	Aa	20	Guerrero et al. (2022)
50.00	−27.70	Pc	62.5	Ayisigi et al. (2020)

Aa *Acidovorax avenae*, At *Agrobacterium tumefaciens*, Cm *Clavibacter michiganensis*, Pc *Pectobacterium carotovorum*, Ps *Pseudomonas syringae*, Pst *Pseudomonas syringae* pv. tomato, Rs *Ralstonia solanacearum*, Xc *Xanthomonas campestris*, Xoo *Xanthomonas oryzae* pv. oryzae, Xap *Xanthomonas axonopodis* pv. punicae, Xv *Xanthomonas vesicatoria*, Xf *Xylella fastidiosa*

*Argirium*-*SUNCs* have an even broader spectrum of action as Molina-Hernandez et al. (2023) documented that it shows strong antimicrobial activity (< 1 ppm) against *Aspergillus niger*, a fungus producing mycotoxins in food and feed (Nielsen et al. 2009). Both in bacteria and in fungi, the main biological target of our nanomaterial is the cell membrane whose depolarization and subsequent loss of function lead to bacterial and fungal death (Molina-Hernandez et al. 2021, 2023). It is worth noting that Ag^+^ in the form of AgNO_3_ does not change the membrane potential (Molina-Hernandez et al. 2023). Therefore, the novelty that characterize *Argirium*-*SUNCs* respect any other nanomaterial is the presence of stable Ag^2+^ and Ag^3+^ cation forms. Membrane depolarization is one of the observed causes of silver nanoparticle antimicrobial activity (Molina-Hernandez et al. 2021; Xu et al. 2020). In fact, when in ionic form (Ag^+^), silver can modify interactions with sulfhydryl groups on the pathogen membrane, obstructing the hydrogen binding sites. Consequently, this affects cellular respiration and electron transfer, inducing a modification in membrane potential. This alteration leads to a loss of membrane integrity, cell lysis, and potentially culminates in cell death (Barras et al. 2018). Through a transcriptomic approach carried out on the *P. syringae* pv. tomato strain used in the study of Orfei et al. (2023a), whose genome we recently sequenced (Orfei et al. 2023b) to better perform the transcriptomic analysis, we demonstrated that the exposition of bacterial cells for 10 and 30 min at sub-letal doses of *Argirium*-*SUNCs* induced change in the expression of genes associated with transporters (*e*.*g*., CadA P-type ATPase, the arsenite resistance efflux pump, multidrug resistance proteins), iron homeostasis (*e*.*g*. FecR, TonB-dependent and EfeUOB transporters), and stress response and primary and nitrogen metabolism (unpublished results).

## *Argirium*-*SUNCs* potential in agriculture

Nanomaterials are receiving increasing interest in agriculture for their contribution in boosting the food crop yield with nanofertilizers and controlling pests and phytopathogens with nanopesticides and nanosensors (Singh et al. 2021). For instance, the use of nanomaterials can render more efficient the delivery mechanisms (Rodrigues et al. 2017; Lowry et al. 2019). Agrochemical delivery is a relevant aspect in sustainable agriculture. The use of agrochemicals is notoriously inefficient as, for example, a large fraction of the 2.5 million tons of pesticides applied per year are either lost to the air and run-off or unable to effectively reach target (Rodrigues et al. 2017). In this context, Orfei et al. (2023a) demonstrated that application of *Argirium*-*SUNCs* during the hydroponic grown of tomato significantly protected leaves from bacterial speck disease caused by *P. syringae* pv. tomato. This suggests that *Argirium*-*SUNCs* are able to enter into the plants via roots and exerts its antimicrobial activity reaching the leaf apoplast where *P. syringae* pv. tomato grown. Root uptake of AgNPs via the apoplastic and symplastic pathways has been well documented (Chavez Soria et al. 2017; Huang et al. 2022) also in tomato plants (Noori et al. 2020). Our results can therefore be exploited for the protection of tomato grown in hydroponics, a system very spread for tomato cultivation (Morgan 2008). The indirect evidence that *Argirium*-*SUNCs* entry into tomato plants via roots leads us to suppose that its protective effect can be also exerted against other tomato bacterial diseases, such as those caused by *Xanthomonas vesicatoria* and *Clavibacter michiganensis*, pathogens against whom it was demonstrated the antimicrobial activity of *Argirium*-*SUNCs *in vitro (Orfei et al. 2023a). *P. syringae* pv. tomato, *X. vesicatoria*, and *C. michiganensis* are seed-transmitted bacteria (Thind 2019) and through infected tomato seeds can be introduced in greenhouses where hydroponic systems are used. Orfei et al. (2023a) also demonstrated that *Argirium*-*SUNCs* strongly inhibited in vitro *Xylella fastidiosa*, a xylem-limited bacterium which re-emerged as a plant pathogen of global importance in 2013 when it was first associated with an olive tree disease epidemic in Italy (Sicard et al. 2018; Saponari et al. 2019). *Argirium*-*SUNCs* could be applied to olive trees through endo-therapy, that is the systemic delivery of active ingredients via trunk injection: a technology that holds promise of a true step change in sustainable olive crop management (Grandi et al. 2023). Endo-therapy allows reaching vascular diseases inaccessible to foliar treatments and delivers active ingredients in a precise manner with no risks of off-target drifts (Grandi et al. 2023). *Argirium*-*SUNCs* could also be applied to plants as seed dressing for its documented growth stimulation activity during tomato seed germination (Orfei et al. 2023a). It is known that delayed and uneven emergence poses a serious problem in the production of horticultural crops, particularly during drought and under adverse weather conditions (Grzesik et al. 2012).

Although spraying agrochemicals directly onto plant leaves is an effective and economic approach in agricultural management (Huang et al. 2022), foliar application of *Argirium*-*SUNCs* to tomato leaves did not protect tomato plants against *P. syringae* pv. tomato (Orfei et al. 2023a). To explain this failure, we hypothesize that foliar application prevents the nanomaterial from reaching the target (intercellular space where *P. syringae* pv. tomato colonize the host) in sufficient quantity to be effective. The very small size of *Argirium*-*SUNCs* (< 2 nm) would justify its entry through the cuticle of the leaves, whose pores are about < 5 nm (Schwab et al. 2016) in size and about 2 nm (Huang et al. 2022). After passing the cuticle, *Argirium*-*SUNCs* could follow the symplastic and/or the apoplastic pathways. In case of the symplastic pathway, the nanomaterial should cross the cell wall and the plasma membrane, through endocytosis and/or non-endocytic pathway, for reaching the cytoplasm and move from cell to cell via plasmodesmata (Stegemeier et al. 2015; Avellan et al. 2019, 2021; Huang et al. 2022). The presence of AgNPs inside plant cells and plasmodesmata has been documented in *Arabidopsis* (He et al. 2022) and lettuce (Larue et al. 2014) plants.

When inside the plant cells, nanomaterials could undergo physical or chemical transformation such as aggregation, oxidative dissolution, chlorination, sulfidation, and complexation with organic ligands (*e*.*g*., glutathione, cysteine), as reported for other AgNPs (Huang et al. 2022). Therefore, these possible transformations of AgNPs inside the plants could prevent the *Argirium*-*SUNCs* from reaching its target.

In the case of apoplastic pathway, *Argirium*-*SUNCs* could move through the cell wall and middle lamella considering that its size is smaller than that of the cell wall and middle lamella pores and from here move to the intercellular spaces (Stegemeier et al. 2015). The presence of AgNPs in cell wall and middle lamella has been documented in *Arabidopsis* (Bao et al. 2016) and tobacco (Cvjetko et al. 2018) plants. It is possible that cell wall retains *Argirium*-*SUNCs* preventing it to reach the optimal concentration to carry out its antimicrobial activity. As documented by He et al. (2022), AgNPs could reach the intercellular spaces and enter from the stomata. Similar to the results obtained with ZnONPs in rice plants (Khan et al. 2021), we can hypothesize that *Argirium*-*SUNCs* are not able to enter through the stomata because it provokes their closure.

Before being placed on market, a new plant protection product must be registered, evaluated, and authorized and toxicological and ecotoxicological documentations are essential aspects in this process (Grillo et al. 2021). In vitro studies carried out on human cell lines (HEK-293, HaCaT, and HMEC) and preclinical one on *Galleria mellonella* indicate that *Argirium*-*SUNCs* are about ten times less toxic in human cells or in *G. mellonella* larvae than that in bacteria and fungi (Pompilio et al. 2018; Grande et al. 2020; Gasbarri et al. 2021; Orfei et al. 2023a; Molina-Hernandez et al. 2023). In addition, *Argirium*-*SUNCs* are no phytotoxic to tomato plants up to 10 ppm, a concentration about ten times less toxic than in phytopathogenic bacteria attacking tomato plants (Orfei et al. 2023a).

## Concluding remarks and future prospects

Chemical control through the use of copper compounds and antibiotics is still an important pillar in controlling bacterial plant diseases worldwide as many of the alternatives to chemical control, when available, are still far from their application. For example, disease resistance through genome editing, which is considered the most effective and eco-friendly measure against bacterial diseases (Sharma et al. 2022), encountered many difficulties in acceptance by consumers (Ishii and Araki 2016). In this context, the developments in the field of nanosized active ingredients and formulations have opened up new avenues for enhancing the delivery and efficacy of pesticides and other agrochemicals (EFSA Scientific Committee 2018). Kah et al. (2018) demonstrated that the effectiveness of the so-called nanopesticides is 20–30% higher respect to the same active ingredient conventionally formulated.

Before marketing and use, a nanopesticide will have to go through stringent regulatory approvals in most countries, and currently, no nanopesticide is listed in the database of active substances approved for use as pesticide in Europe (Grillo et al. 2021). The road to authorization is long and bumpy also because (i) there is no agreement on the definition of nanopesticide; (ii) Scientific Advisory Panel of the US Federal Insecticide, Fungicide, and Rodenticide Act (FIFRA) believe that the environmental risks of nanopesticides may be different from conventional pesticides, and current methods may not be adequate to remediate or predict the environmental fate and effects (Grillo et al. 2021). The European Food Safety Authority (EFSA) considered the term nanopesticide as a synonym for plant protection products (PPP) in a broad way (EFSA Scientific Committee 2018), and PPP are mainly regulated by Regulation (EC) No 1107/2009 before being placed in the market. According to this European Regulation, procedure and criteria for the approval of active substances include scientific investigations on the efficacy, the methods of analysis, the impact on human health, the fate and behavior in the environment, and the ecotoxicology. In a possible registration, evaluation, and authorization process of *Argirium*-*SUNCs* as a product for protecting plants from bacterial diseases, we can consider that (i) its efficacy in vitro is very high. The MIC value of *Argirium*-*SUNCs* for *Pseudomonas syringae* pv. tomato is 625 times lower than that of Kocide 3000 (Li et al. 2017; Orfei et al. 2023a), a commercial product containing copper hydroxide as active ingredient, the most used a.i. in the world in protecting plants from bacterial diseases. (ii) The methods of analysis of AgNPs are well documented (Wang et al. 2020). (iii) There are inconsistent reports about the toxicity of engineered nanomaterials on human health typically influenced by several factors that can impact the toxicity study (Asmatulu et al. 2022). These factors include the type of cell line, type of nanomaterial, functionalization, synthesis process of the nanomaterials, dosage, size, method of mixing, exposure method, surface charge, shape, gender of the animal model, and cell medium, thus making it exceedingly complicated to determine the risk of engineered nanomaterials or determine their impact on humans (Asmatulu et al. 2022). (iv) The fate and behavior in the environment of AgNPs has been studied (Fabrega et al. 2011); ecotoxicological studied on AgNPs have been reported (Courtois et al. 2019).

Further studies are necessary to investigate *Argirium*-*SUNCs* on the modality of delivery on the doses and timing of treatments.

## Data Availability

Not applicable
